# LY294002 Is a Promising Inhibitor to Overcome Sorafenib Resistance in FLT3-ITD Mutant AML Cells by Interfering With PI3K/Akt Signaling Pathway

**DOI:** 10.3389/fonc.2021.782065

**Published:** 2021-11-08

**Authors:** Amin Huang, Peiting Zeng, Yinguang Li, Wenhua Lu, Yaoming Lai

**Affiliations:** ^1^ Department of Medical Oncology of the East Division, The First Affiliated Hospital, Sun Yat-Sen University, Guangzhou, China; ^2^ State Key Laboratory of Oncology in Southern China, Sun Yat-Sen University Cancer Center, Guangzhou, China; ^3^ Department of Obstetrics and Gynecology of the East Division, The First Affiliated Hospital, Sun Yat-Sen University, Guangzhou, China; ^4^ Department of Rehabilitation, The Fifth Affiliated Hospital of Guangzhou Medical University, Guangzhou, China

**Keywords:** FMS-like tyrosine kinase 3, Internal tandem duplication, LY294002, Drug resistance, Acute myeloid leukemia, PI3K/Akt

## Abstract

Internal tandem duplications (ITD) mutation within FMS-like tyrosine kinase 3 (FLT3), the most frequent mutation happens in almost 20% acute myeloid leukemia (AML) patients, always predicts a poor prognosis. As a small molecule tyrosine kinase inhibitor, sorafenib is clinically used for the treatment of advanced renal cell carcinoma (RCC), hepatocellular carcinoma (HCC), and differentiated thyroid cancer (DTC), with its preclinical and clinical activity demonstrated in the treatment of Fms-like tyrosine kinase 3-internal tandem duplication (FLT3-ITD) mutant AML. Even though it shows a rosy future in the AML treatment, the short response duration remains a vital problem that leads to treatment failure. Rapid onset of drug resistance is still a thorny problem that we cannot overlook. Although the mechanisms of drug resistance have been studied extensively in the past years, there is still no consensus on the exact reason for resistance and without effective therapeutic regimens established clinically. My previous work reported that sorafenib-resistant FLT3-ITD mutant AML cells displayed mitochondria dysfunction, which rendered cells depending on glycolysis for energy supply. In my present one, we further illustrated that losing the target protein FLT3 and the continuously activated PI3K/Akt signaling pathway may be the reason for drug resistance, with sustained activation of PI3K/AKT signaling responsible for the highly glycolytic activity and adenosine triphosphate (ATP) generation. PI3K inhibitor, LY294002, can block PI3K/AKT signaling, further inhibit glycolysis to disturb ATP production, and finally induce cell apoptosis. This finding would pave the way to remedy the FLT3-ITD mutant AML patients who failed with FLT3 targeted therapy.

## Introduction

Internal tandem duplications (ITD) mutation, the most frequent mutation in the juxtamembrane domain of the FMS-like tyrosine kinase 3 (FLT3) gene, was found in almost 20% of all acute myeloid leukemia (AML) patients (1, 2). This mutation leads to constitutive activation of FLT3 and its downstream signaling, including phosphatidylinositol3-kinase (PI3K)/AKT, mitogen-activated protein kinase/extracellular signal-regulated kinase (MAPK/ERK), and signal transducer and activator of transcription5 (STAT5). It also results in cell proliferation and disease progress (3–5). It is demonstrated that FLT3 mutational status, an independent predictor of poor prognosis, was closely associated with chemotherapy efficacy and disease relapse (6, 7). Many inhibitors targeting FLT3 kinase are under development, with some already demonstrating their efficacy in treating FLT3-ITD mutant AML (8–10).

Sorafenib, firstly known for its antiangiogenic effect and approved for treating HCC, RCC and DTC, is found potent in inhibiting FLT3, with a significant anti-tumor effect in the FLT3-ITD mutant AML (11–13). Clinical trials of sorafenib were widely conducted in the first-line induction, relapsed or refractory and post-transplant maintenance period, as a single agent, and in combination with chemotherapy and hypomethylating agents. Farhad et al. reported the combination of sorafenib and chemotherapy (cytarabine and idarubicin) resulted in a higher CR rate in FLT3-mutated patients than that of the FLT3-WT patients (14). However, no significant difference in overall survival (OS) or disease-free survival (DFS) was observed in the final analysis due to the emergence of sorafenib-resistant leukemic clones (15). SORAML, a phase 2 trial, investigated the effect of sorafenib in consolidation therapy and maintenance. Median event-free survival in the sorafenib group was 21months versus 9 months in the placebo group. The combination effect of sorafenib with azacitidine in older patients unsuitable for intensive induction therapy was evaluated. In the 27 patients enrolled, 7 patients reached CR, 12 CRi/CRp, and 2 PR, with an overall response rate, 78% (16). As monotherapy, sorafenib has been proven effective in the salvage therapy for R/R FLT3-ITD AML and maintenance after transplantation by reducing the possibility of relapse and death (17, 18). Besides sorafenib, other FLT3 inhibitors, such as midostaurin, gilteritinib and quizartinib were also approved by Food and Drug Administration (FDA) for treating FLT3 mutant AML (19–22). Both European LeukemiaNet and National Comprehensive Cancer Network guidelines recommend FLT3 genetic testing to predict the prognosis and evaluate the targeted therapy when the disease is diagnosed and relapsed.

Eventhough FLT3 inhibitors are promising in treating FLT3-ITD mutant AML patients, the limitations should not be ignored: drug resistance that often occurs within 1–3 months after initial remission responses, resulting in disease relapse and treatment failure (23). As massive studies have investigated the resistance mechanism, multiple mechanisms have been proposed, including increased efflux of drugs induced by ATP-binding cassette (ABC) proteins, the emergence of new mutations, up-regulation of FLT3 ligand, aberrant activation of pro-survival signaling, rewired metabolic profiles and redox change (24–32). However, there is still no consensus on the precise mechanisms of drug resistance, and more efforts should be taken in clarifying the mechanisms and exploring new strategies to reverse this resistance.

In my previous paper, we established two sorafenib-resistant cell lines and compared the metabolic differences between the resistant cells and their corresponding parental cells. We found the resistant cells were with mitochondria dysfunction and active aerobic glycolysis (30). In my present work, we will further elucidate the relationship between PI3K/Akt signaling and glycolysis and investigate the possibility of overcoming this resistance by interfering PI3K/Akt signaling pathway.

## Materials and Methods

### Cell Lines and Cell Culture

Human FLT3-ITD mutant cell line: MV4-11 was ordered from ATCC. BaF3-ITD cell line was established as described previously from mouse hematopoietic progenitor BaF3 cell line (33). All parental cells were cultured in RPMI 1640 with 2.05 mM L-Glutamine (Hyclone, South Logan, UT, USA) and 10% Fetal Bovine Serum (Hyclone, South Logan, UT, USA) in the incubator with 5% CO2 at 37°C.

### Generation of the Drug-Resistant Cell Lines

To generate drug-resistant cell lines, we co-cultured the BaF3-ITD and MV4-11 cells with sorafenib with the initial concentration of 1 nM. When the cells could survive in the present concentration for 3-5 days, the concentration of sorafenib would be improved to a higher dose. Three months later, the resistant cell lines, MV4-11-R and BaF3-ITD-R cell lines could grow in a medium containing 0.5µM sorafenib. The sorafenib-resistant cells were continuously cultured in the RPMI 1640 medium with 0.5 µM sorafenib.

### Chemical Compounds and Biologic Reagents

All small molecule inhibitors were obtained from Selleck Chemicals (Houston, TX, USA), these inhibitors include: Sorafenib tosylate (#S1040), PI3K inhibitors: LY294002 (#S1105), Buparlisib (#S2247), Pictilisib (#S1065), Alpelisib (#S2814) and Akt inhibitors MK2206 (#S1078), Ipatasertib (#S2808), Afuresertib (#S7521). All chemicals formulated by their suppliers’ recommendations.

### Antibodies

Antibodies of anti–HK2 (#2867), anti–PKM2 (#4053), anti–P-FLT3 (#3464), anti–FLT3 (#3462), anti–Akt (#4691), anti–P-Akt (#4060), anti–PARP (#9542) were obtained from Cell Signaling Technologies (Beverly, MA, USA); Antibodies of anti–PDK1 (#ab11025), anti–β-actin (#ab8227), goat anti-Rabbit secondary HRP-conjugated antibody (#ab6721), and goat anti-Mouse secondary HRP-conjugated antibody (#ab6789) were supplied by Abcam (Cambridge, UK).

### Cell Proliferation Assays

Cell viability assay was performed using CellTiter 96 AQueous One Solution Reagent (Promega, Madison, WI, USA) according to the manufacturer’s recommendations. Briefly, cells were plated in 96-well microplates in triplicate at a density of 3×10^5^cells/ml and treated as indicated for 72 hrs. Then 20 μl of a tetrazolium compound (MTS) was added to each well for 4 h at 37°C. After incubation, the absorbance was read at a wave length of 490 nm. Accordingly, cell viability is reported as percentage of control (untreated) cells, with the data representing three independent experiments. Error bars represent the standard error of the mean for each data point.

### Western Blot

Protein was extracted by RIPA lysis buffer supplemented with protease inhibitor cocktail set I and phosphatase inhibitor cocktail set II (Calbiochem, Nottingham, England). Equal amounts of protein extracts were resolved in 4%-12% standard SDS-PAGE and transferred to polyvinylidenedifluoride (PVDF) membranes. Membranes were probed with primary antibody overnight at 4°C and Horseradish peroxidase (HRP)-conjugated secondary antibody for 1 h at room temperature, with the protein band visualized by chemiluminescent detection kit with ClarityTM Western ECL Substrate from Bio-Rad Laboratories (Richmond, CA, USA). The images were captured using Bio-Rad ChemiDoc Imaging System.

### Quantitative Real-Time PCR

Ribonucleic acid (RNA) was isolated with TRIzol reagent (Invitrogen, Paisley, United Kingdom), and complementary DNA (cDNA) was generated using PrimeScript RT reagent Kit (Takara Shuzo, Shiga, Japan) according to the manufacturer’s recommendation. Quantitative real-time polymerase chain reaction (PCR) was performed with SYBR Premix Ex Taq II (Takara Shuzo, Shiga, Japan) according to the manufacturer’s recommendation. The quantitative polymerase chain reaction was carried out in triplicate on a CFX96 multicolor real-time PCR system (Bio-Rad, Richmond, CA).β-actin was used as an internal standard. The PCR primers are listed in **Table 1**.

**Table 1:** Specific primer sequences used in this paper (5′-3′).

	Gene name	Forward	Reverse	Accession number
Homo	PKM2	CCACTTGCAATTATTTGAGGAA	GTGAGCAGACCTGCCAGACT	NM_002654
	HK2	CAAAGTGACAGTGGGTGTGG	GCCAGGTCCTTCACTGTCTC	NM_000189
	β-actin	AACTCCATCATGAAGTGTGACG	GATCCACATCTGCTGGAAGG	NM_001101
	LDHA	ATCTTGACCTACGTGGCTTGGA	CCATACAGGCACACTGGAATCTC	NM_005566
	GLUT1	CGGGCCAAGAGTGTGCTAAA	TGACGATACCGGAGCCAATG	NM_006516
	FLT3	CTTCCCTTTCATCCAAGACAACATC	ATCCACATTCTGATACATCGCTTCT	NM_004119
musculus	β-actin	GTGACGTTGACATCCGTAAAGA	GCCGGACTCATCGTACTCC	NM_007392
	flt3	TTTCATCCAAGACAACATCTCCTTC	CTAAAAATGAAGTCAGGTTGGGGAA	NM_010229

### Flow Cytometry

Apoptosis detection kit containing Annexin-V-FITC, propidiumiodide (PI) and binding buffer were purchased from Keygen Biotech (Nanjing, China). To detect apoptosis, cells were seeded in a 6-well plate at the density of 3×10^5^ cells/well and incubated with designated drugs for 48h. The cells were collected and washed with cold phosphate-buffered saline (PBS) three times. Each sample was suspended in 500 µl binding buffer containing 5 µl Annexin-V-FITC, 5 µl PI, and incubated for 15 min in the dark at room temperature. The cells were analyzed by CytoFlex flow cytometry (Becton Dickinson, Heidelberg, Germany) and CytExpert software. The results shown here represent three independent experiments.

### Glucose Uptake and Lactate Production

To determine the cellular glucose uptake and lactate production, cells were collected and seeded in triplicate at the density of 1~2×10^6^ cells/well and treated with certain drugs for indicated times. Culture mediums were removed to analyze glucose and lactate content with the fresh medium as control using a SBA-40C biosensor (Biology institute of Shandong academy of science, Jinan, Shandong province) according to the manufacturer’s instruction.

### Statistical Analysis

The statistical analyses were performed with SPSS version 18.0 software (SPSS Inc., Chicago, IL, USA). Comparisons between groups were analyzed by the Student paired t test. Multiple groups were compared using analysis of variance (ANOVA) followed by post-hoc Fisher’s least significant difference (LSD) testing when appropriate. Significance was judged when p<0.05.

## Results

### Evaluation of the Drug-Resistant Characteristics: Comparison of Proliferation and Apoptosis in the Presence of Sorafenib

**Figure 1:**
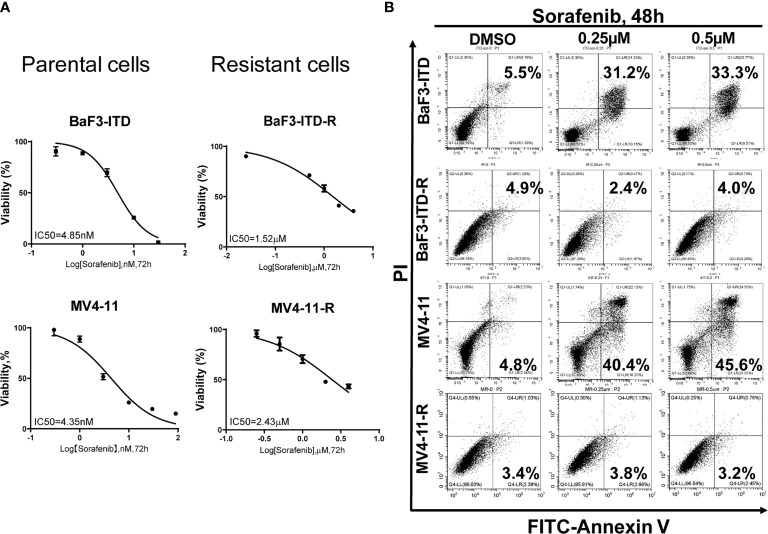
Comparison of the cell proliferation and apoptosis of the parental and sorafenib-resistant cell lines in the presence of sorafenib. **(A)** The sorafenib-resistant cells, MV4-11-R and BaF3-ITD-R, and parental BaF3-ITD and MV4-11 cells were incubated with increasing concentrations of sorafenib for 72 hours. Cell viability (%) was compared. Data represent three independent experiments. **(B)** Cells were exposed to sorafenib at the indicated concentrations for 48 hours. The apoptotic cells were stained with Annexin-V and propidium iodide (PI) for 15 mins and detected by flow cytometry in one hour. Data represent three independent experiments.

Two sorafenib resistant cell lines, MV4-11-R and BaF3-ITD-R, generated from the parental MV4-11 and BaF3-ITD cells should be continuously maintained in the medium containing 0.5 µM sorafenib. When deprived with sorafenib treatment they may lose the drug resistance. After a long time to store in the liquid nitrogen, it’s necessary to confirm the drug resistance prior to use. Cell viability and cell apoptosis assays were utilized to evaluate the drug resistance characteristics. Cell viability of MV4-11 and MV4-11-R cells responding to increasing concentrations of sorafenib for 72 h, and that of BaF3-ITD and BaF3-ITD-R were shown in **Figure 1A**. The IC50 values of the two pairs of cells were evaluated. We estimated the IC50 for resistant cell line MV4-11-R and BaF3-ITD-R is 2.43 μM and 1.52 μM, which is significantly higher than 4.35 nM in MV4-11 and 4.85 nM in BaF3-ITD. By analyzing the cell viability, we confirmed the resistant cell lines were resistant to sorafenib treatment.

Furthermore, flow cytometry was used to evaluate apoptosis when cells were treated with sorafenib. As shown in **Figure 1B**, we didn’t observe any cell apoptosis after incubation MV4-11-R and BaF3-ITD-R cells with 0.25 μM sorafenib for 48h. However, after incubating the parental cells in 0.25 μM sorafenib for 48h, significant cell apoptosis was observed in MV4-11 and BaF3-ITD cells (31.2% and 40.4%, respectively). When the concentration was increased to 0.5 μM, more apoptosis was detected in the parental cells, and still no apoptosis was observed in the resistant cells. From this figure, we further confirmed the resistant cells kept their drug-resistance characteristics after long time of storing in liquid nitrogen.

### The Significant Restraining of FLT3 Is Concurrent With Sustained Activation of PI3K/Akt Signaling in the Resistant Cells

**Figure 2:**
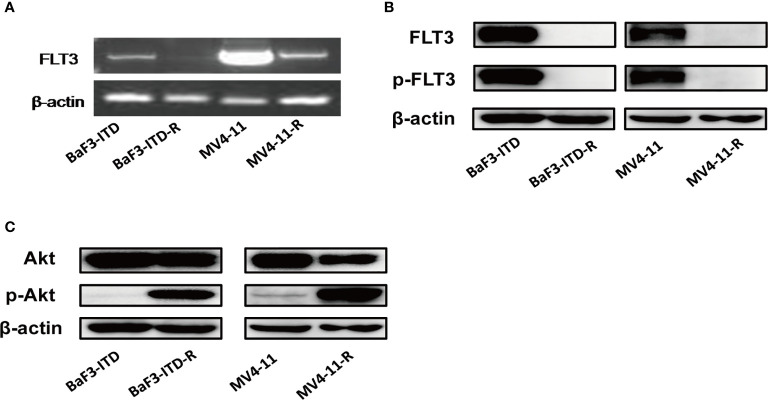
FLT3 and PI3K/Akt signaling were detected. **(A)** PCR product of FLT3 gene was applied to agarose gel electrophoresis. β-actin was used as an internal control. **(B, C)** The protein level of total FLT3, phosphorylated-FLT3, total Akt, phosphorylated-Akt in BaF3-ITD, BaF3-ITD-R, MV4-11, MV4-11-R cells were evaluated by immunoblotting, with β-actin used as aloading control.

Sorafenib induced FLT3-ITD mutant AML cell death by targeting FLT3 and downstream signaling, including PI3K/AKT, MAPK/ERK, and STAT5. Firstly, the FLT3 gene expression examined by PCR and then visualized by agarose gel electrophoresis in **Figure 2A** indicated that FLT3 gene expression was dramatically inhibited by sorafeinb in the resistant cells. Corresponding to the gene expression, FLT3 and phosphorylated FLT3 protein expression were not observed in the sorafenib-resistant cells compared with the parental cells (**Figure 2B**). However, the phosphorylated Akt was remarkablely activated (**Figure 2C**), indicating the lost target protein may be responsible for drug resistance to FLT3 inhibitors. However, PI3K/Akt signaling’s sustained activation may provide survival advantages for the resistant cells.

### The Resistant Cells Rely on Glucose for Proliferation

**Figure 3:**
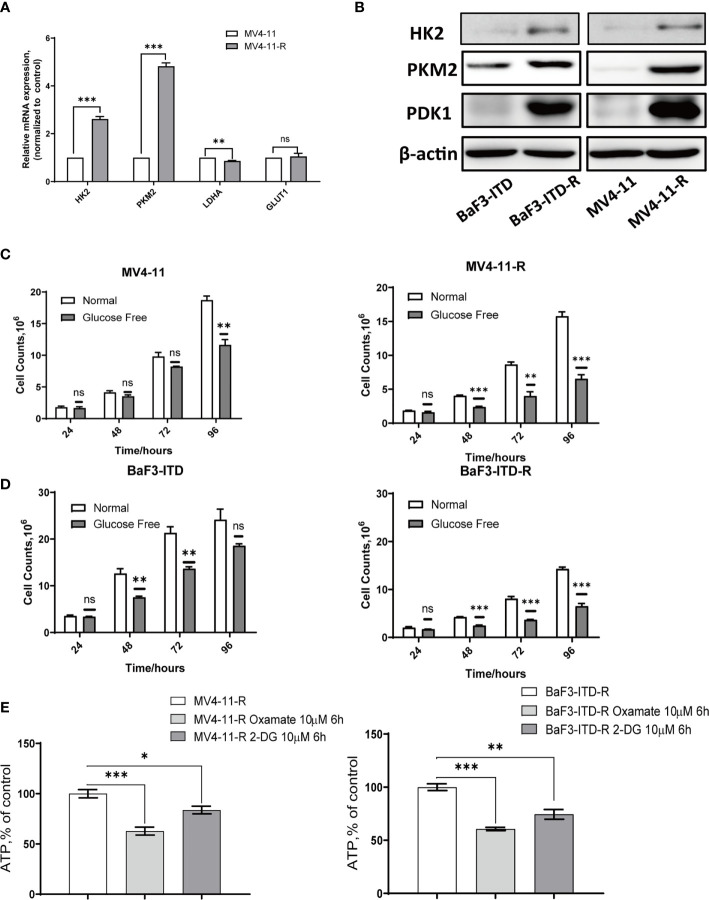
Sorafenib-resistant cells rely on glucose for proliferation and ATP generation. **(A)** Real-time quantitative PCR was used to compare the gene expression of glycolytic enzymes. **(B)** Western blot analysis of glycolytic enzyme HK2, PKM2 and PDK1. β-actin was shown as a loading control. **(C, D)** Cell counts were compared for the two pairs of cells after incubating with the normal and glucose-free medium for indicated times. **(E)** ATP production rate of the resistant cells was detected when treated with 10 µM Oxamate and 10 µM 2-DG, respectively, for 6 hours. *p < 0.05, **p < 0.01, ***p < 0.001, NS, No significance.

The activation of the PI3K/Akt signaling pathway is known to regulate cell death and survival, and enhance glycolytic activity and metabolism (34). Plentiful work had shown that increased Akt activation could directly phosphorylate a number of glycolytic enzymes such as hexokinase 2 (HK2) (35). Based on the hyper-activation of PI3K/Akt signaling, we firstly examined glycolytic genes, including HK2, pyruvate kinase isozyme M2 (PKM2), lactate dehydrogenase A (LDHA), and glucose transporter type 1 (GLUT1), found HK2, PKM2 and GLUT1 genes were over-expressed in varying degrees in the resistant cells. Western blotting further confirmed the up-regulation of HK2, PKM2 and 3-phosphoinositide dependent kinase-1 (PDK1) in protein level, as shown in **Figures 3A, B**. When the glucose in the medium was deprived, the proliferation of resistant cells was inhibited more significantly than that of the corresponding parental cells, especially for MV4-11 cells, so deprivation of glucose indeed inhibited cells growth from 24h to 72h. However, this inhibition didn’t show any statistical significance (P>0.05). However, this difference was more apparent for the MV4-11-R cells, with the cell growth suppressed significantly from 48h, as shown in **Figure 3C** (P<0.001). For BaF3-ITD and BaF3-ITD-R cell lines, same phenomenon was observed, as shown in **Figure 3D**, the inhibition effect was more apparent for the resistant cells at 48 h, 72 h, and 96h. That means the resistant cells are more dependent on glucose for cell proliferation and energy supply. Glycolysis is the major way of adenosine triphosphate (ATP) production for the resistant cells since mitochondria dysfunction was reported in my previous work (26). Intracellular ATP was measured when the enzymes relevant to glucose metabolism were interfered. By treating the resistant cells with 10 μM oxamate, a competitive LDHA inhibitor, for 6 hrs, we observed the ATP production was reduced to 62.8% and 60.7% for MV4-11-R and BaF3-ITD-R respectively (P<0.001). 2-deoxy-d-glucose (2-DG), a derivative of glucose, can be phosphorylated by hexokinase and interfere with glucose uptake. Similar phenomenon was observed when cells were treated with 10μM 2-DG for 6 hrs, with ATP production significantly reduced to 83.7% and 74.7% for MV4-11-R and BaF3-ITD-R, respectively (P<0.05) (**Figure 3E**). These results mean the resistant cells depended on glycolysis for survival and ATP production.

### PI3K/Akt Signaling Was Responsible for the Highly Activated Glycolytic Activity

**Figure 4:**
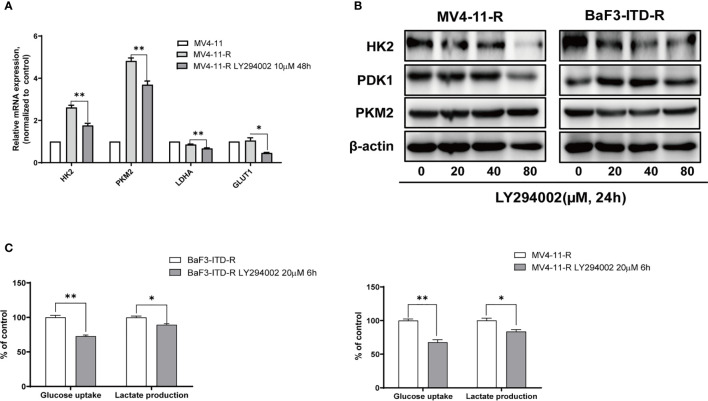
Gene expression of glycolytic enzymes and ATP generation were diminished by LY294002 in the resistant cells. **(A)** Incubating the MV4-11-R cells with 10 μM LY294002 for 48 hours, and messenger ribonucleic acid (mRNA) level of HK2, PKM2, LDHA, GLUT1 were compared. **(B)** The sorafenib-resistant cells, MV4-11-R and BaF3-ITD-R, were incubated with increasing concentrations of LY294002 for 24 hours, with the protein expression of HK2, PDK1 and PKM2 were evaluated by western blotting. β-actin was used as a loading control. **(C)** Glucose uptake and lactate production were evaluated when resistant cells were treated with 10µM LY294002 for 6 hours.*p < 0.05, **p < 0.01.

To illustrate whether the highly expressed glycolytic enzymes were associated with retained activation of PI3K signaling, a pan-PI3K inhibitor, LY294002 was applied to treat the resistant cells. The real-time quantitative polymerase chain reaction (PCR) revealed that when the cells were treated with 20 μM LY294002 for 48 h, gene expression of HK2, PKM2 and Glut1 were obviously depleted (**Figure 4A**). Western Blot also verified the protein expression inhibition of glycolytic enzymes induced by LY294002 (**Figure 4B**). As the concentration of LY294002 increased, HK2 were significantly suppressed in both MV4-11-R and BaF3-ITD-R cells, however PDK1 and PKM2 level were not apparently influenced by LY294002 treatment. Moreover, co-culturing cells with 20 μM LY294002 for only 6 hrs, glucose uptake could be reduced to 72.8% and 73.5% for BaF3-ITD-R and MV4-11-R, respectively (P<0.01), with their lactate production inhibited to 89.2% and 85.3% (P<0.05) (**Figure 4C**). Thus, we speculate activation of PI3K/Akt axis maybe the incentive factor for the highly glycolysis of the drug-resistant cells and the crucial pathway for cell survival and proliferation. This leads us to investigate further whether blocking this pathway could be the potential therapeutic regimen to conquer drug-resistance.

### The Resistant Cells Were More Sensitive to PI3K Inhibitor LY294002

**Figure 5:**
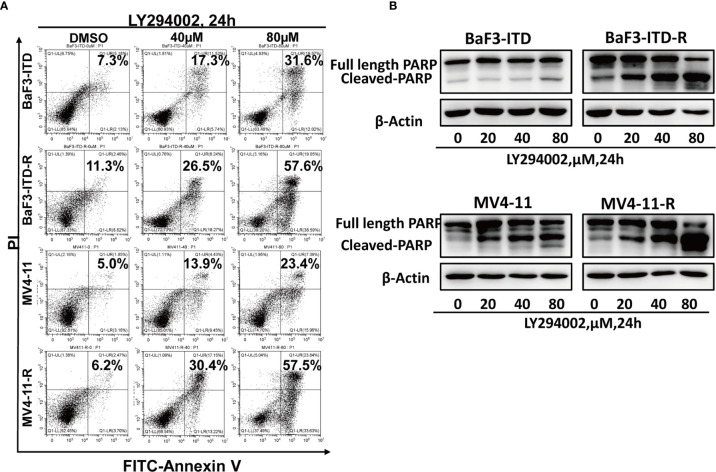
Drug-resistant cells are more sensitive to LY294002. **(A)** Comparison of the apoptotic effect induced by LY294002 in two pairs of cell lines. Cells were exposed to LY294002 at the indicated concentrations for 24 hours, and the apoptotic cells were stained with Annexin-V and PI for 15 mins and detected by flow cytometry in one hour. Data represent three independent experiments. **(B)** Cells were treated with LY294002 for increasing concentrations, with PARP and cleaved PARP detected by western blotting. β-actin was used as a loading control.

As a highly glycolytic activity of resistant cells is promoted by the activation of PI3K/Akt signaling, interfering with this signaling pathway can suppress glycolysis and cell growth. So, we speculated PI3K inhibitor could selectively induce resistant cell death and reverse drug resistance. To verify this hypothesis, the pan PI3K inhibitor LY294002 was adopted to treat drug-resistant cells. LY294002 could induce cell apoptosis for both the drug-resistant and the parental cells in a dose-dependent manner, but the apoptosis was more significant in the resistant cells. As illustrated in **Figure 5A**, in the presence of 40 µM LY294002 for 24h, the apoptotic rate of BaF3-ITD-R cells was 26.5%, while for the BaF3-ITD cells, the value was only 17.3%. When the concentration reached to 80 µM, 57.6% of apoptosis was observed in the resistant cell BaF3-ITD-R. However, the value was only 31.6% for the BaF3-ITD cells. The same phenomenon was observed in MV4-11 and MV4-11-R cells, with LY294002 inducing more apoptosis in the resistant cells than parental cells. Poly-ADP-ribose polymerase (PARP) and cleaved PARP were detected by western blotting to confirm the cell apoptosis induced by LY294002. As shown in **Figure 5B**, inhibition of PI3K by LY294002 induced the appearance of cleaved PARP in a dose-dependent manner. Treatment of BaF3-ITD and BaF3-ITD-R cells with 20 µM LY294002 for 24h witnessed prominent up-regulation of cleaved PARP in the BaF3-ITD-R cells, yet no obvious change was observed until the concentration comes to 80 µM, only slight change was detected for the BaF3-ITD cells. Similar results were observed in the MV4-11 and MV4-11-R cells. When cells were treated with 80 µM LY294002 for 24h, more apparent cleaved PARP was detected in the MV4-11-R cell line than that of MV4-11 cells. These results mean that the drug-resistant cells were much more sensitive to PI3K inhibitor, and LY294002 is promising in overcoming FLT3 inhibitor induced drug-resistance. We also evaluated the drug sensitivity of the other PI3K and Akt inhibitor, as shown in **Supplementary Figure 1**, we found the drug resistance cells showed resistant to all these inhibitors in different degrees. This means that it’s not all the PI3K/Akt pathway inhibitors could overcome the drug resistance. As a PI3K inhibitor, LY294002 has its special function and mechanism that deserve further investigation.

## Discussion

Internal tandem duplication of FLT3, the predictor of poor prognosis, occurs in a subset of patients with AML. Although various FLT3 inhibitors have been developed, with several applied to clinical practices, the development of drug resistance remains a major challenge. The mechanisms of drug resistance have been extensively studied, and efforts are underway to develop new strategies to overcome this resistance. However, no effective treatment procedure is found and approved clinically.

In this paper, we established two sorafenib-resistant cells by culturing BaF3-ITD and MV4-11 cells with increasing concentrations of sorafenib for almost three months. By comparing the resistant cells with their parental ones, we tried to investigate mechanisms of sorafenib resistance in human leukemic cell lines. The drug-resistance was firstly determined by calculating the IC50 value and detecting the cell apoptosis in the presence of sorafenib, with the resistant cells confirmed, highly resistant to sorafenib treatment. FLT3 is the target of FLT3 inhibitors. Western blot analysis revealed that prolonged sorafenib treatment resulted in significant down-regulation of total and phosphorylated FLT3 with the retained downstream signaling PI3K/Akt activated. My previous study also reported the resistant cells were cross-resistant to FLT3 inhibitor, protein kinase C 412 (PKC412) and tandutinib (MLN518) (30). That means the lost target protein FLT3 leads to ineffective of FLT3 inhibitors. However, sustained activation of PI3K/Akt pathway resulted in cell proliferation and survival. These together possibly explain the underlying mechanism for drug resistance.

The PI3K/Akt signaling pathway plays a vital role in regulating cell proliferation, survival, and apoptosis (36). Moreover, it is closely relevant to aerobic glycolysis through interacting with glycolytic enzymes. As the enzyme controlling glucose uptake into cells, GLUT1, regulated by Akt, can enhance glucose uptake. HK2, the rate-limiting enzyme in glycolysis, sequesters glucose inside the cells by phosphorylating it to glucose-6-phosphate, with Akt activation promoting HK2 localized to mitochondria. This process leads to rapid mitochondria-derived ATP production and sustained HK2-mediated glucose phosphorylation (35, 37). The downstream effector of Akt, mammalian target of rapamycin (mTOR), was reported to enhance PKM2 expression by simulating HIF1α expression (38). Besides, PI3K/Akt could also stimulate the activity of other enzymes such as phosphofructokinase 1 (PFK1) and PDK1 (36, 39).

Based on this, we speculated the resistant cells with highly activated PI3K/Akt signaling may have more active glycolytic activity. By qRT-PCR and western blotting, we found the resistant panels were over-expressed with PKM2 and HK2. Furthermore, when culturing cells in the medium deprived of glucose, more significant growth inhibition were observed in the drug-resistant cells than the parental. That means the resistant cells were more dependent on glucose for proliferation. Blocking the glycolytic pathway, with 2-DG and oxamate could sharply shrink ATP production and cell survival.

To further illustrate the relationship between PI3K/Akt signaling and glycolysis, PI3K inhibitor, LY294002, was adopted to treat cells. The discovered suppression of glycolytic enzymes, glucose uptake and lactate production represent the inhibition of the glycolytic pathway. Cell apoptosis assay and PARP cleavage detected by western blotting further verified the cytotoxicity of LY294002.

In summary, through prolonged exposure to sorafenib, we established two pairs of drug-resistant cells. We demonstrated that drug-resistant cells, losing the target protein FLT3, lead to resistance to TKI inhibitors. The continuous activation of PI3K/Akt signaling, the major downstream signaling of FLT3, led to highly glycolytic activity that provides ATP production and cell survival advantages. As PI3K/Akt signaling is the initiator for metabolic changes and cell survival, we hypothesized that blocking this pathway with PI3K inhibitors may selectively induce cell death and conquer drug resistance. Further experiments confirmed this speculation, and PI3K inhibitor LY294002 may provide a new therapeutic regimen to combat sorafenib-induced drug resistance. In the meanwhile, the other PI3K and Akt inhibitors were tested for their drug sensitivities, the result showed the BaF3-ITD-R cells were resistant to all these inhibitors. This leads us to speculate that besides inhibiting PI3K, LY294002 may have other mechanisms to interfere with the survival of resistant cells. The underlying mechanisms need further investigations. Moreover, the anti-tumor effect of LY294002 should be investigated in animal experiments and possibly in clinical trials in future.

## Data Availability Statement

The original contributions presented in the study are included in the article/**Supplementary Material**. Further inquiries can be directed to the corresponding author.

## Author Contributions

AH designed the research study. AH, WL, PZ, and YLi conducted the cell experiments. AH, YLi, and YLai were involved in data analysis. AH was responsible for writing the manuscript. All authors contributed to the article and approved the submitted version.

## Funding

This work was supported by National Natural Science Foundation of China (No.81900150), Medical Scientific Research Foundation of Guangdong Province, China (No. A2019451), The Science and Technology Planning Project of Guangzhou, China (No. 202102020741).

## Conflict of Interest

The authors declare that the research was conducted in the absence of any commercial or financial relationships that could be construed as a potential conflict of interest.

The reviewer LZ declared a shared parent affiliation with one of the authors, YLa, to the handling editor at time of review.

## Publisher’s Note

All claims expressed in this article are solely those of the authors and do not necessarily represent those of their affiliated organizations, or those of the publisher, the editors and the reviewers. Any product that may be evaluated in this article, or claim that may be made by its manufacturer, is not guaranteed or endorsed by the publisher.

## Supplementary Material

Supplementary Figure 1 Effect of the other PI3K and Akt inhibitor in the proliferation of BaF3-ITD and BaF3-ITD-R cells. BaF3-ITD and BaF3-ITD-R cells were incubated with increasing concentrations of the other PI3K inhibitor: buparlisib, pictilisib, alpelisib and Akt inhibitors: afuresertib, MK2206, ipatasertib for 72 hours. Cell viability (%) was detected by MTS method.
