# Supplementary figures and images for: LY294002 Is a Promising Inhibitor to Overcome Sorafenib Resistance in FLT3-ITD Mutant AML Cells by Interfering With PI3K/Akt Signaling Pathway
Source: Front Oncol. 2021 Nov 8;11:782065. doi: 10.3389/fonc.2021.782065 (PMC8606661; doi:10.3389/fonc.2021.782065)

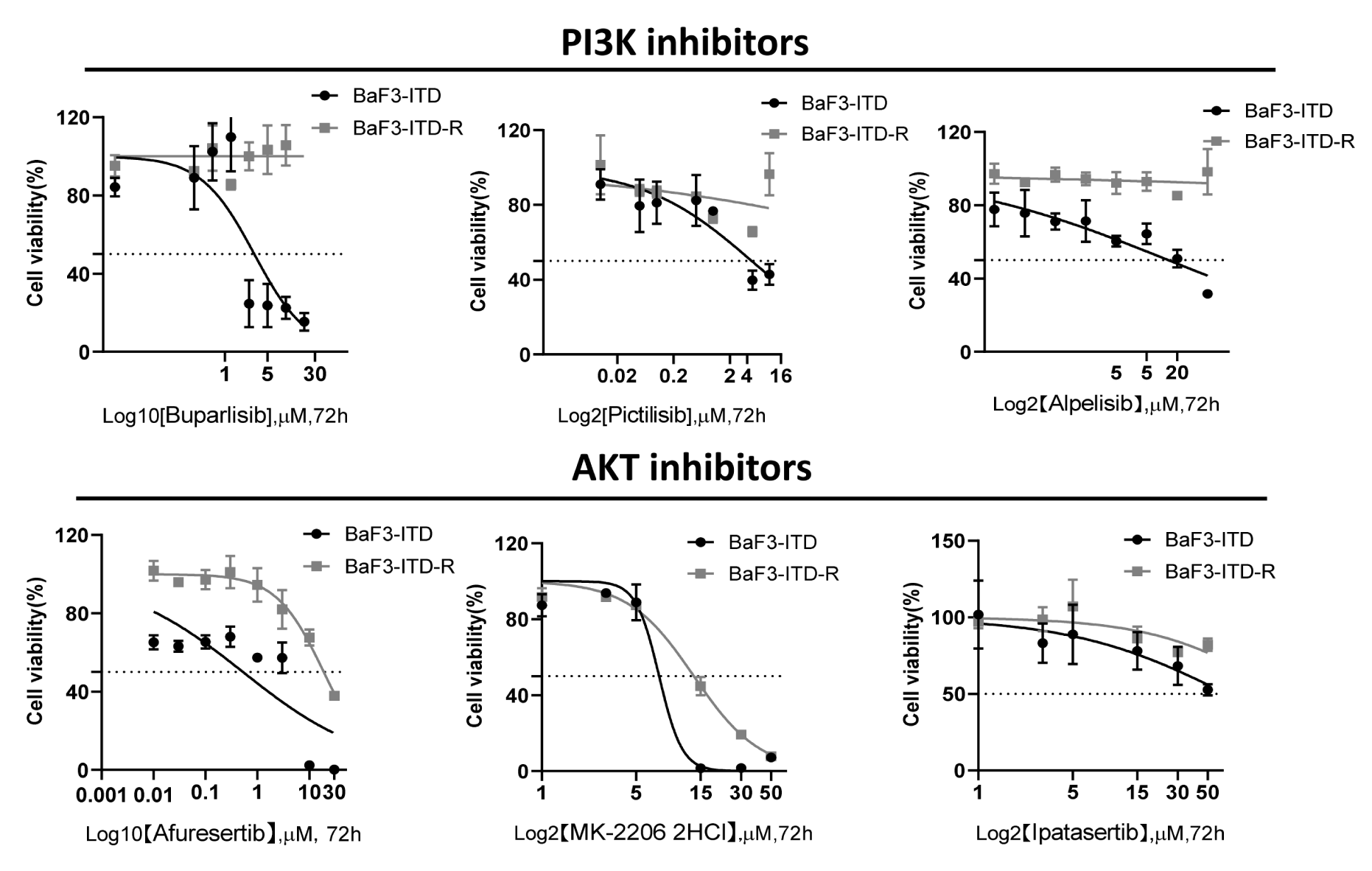

Supplement: Supplementary Figure 1 — Effect of the other PI3K and Akt inhibitor in the proliferation of BaF3-ITD and BaF3-ITD-R cells. BaF3-ITD and BaF3-ITD-R cells were incubated with increasing concentrations of the other PI3K inhibitor: buparlisib, pictilisib, alpelisib and Akt inhibitors: afuresertib, MK2206, ipatasertib for 72 hours. Cell viability (%) was detected by MTS method. [file Image_1.tif]
